# Federated Semi-Supervised Learning with Uniform Random and Lattice-Based Client Sampling

**DOI:** 10.3390/e27080804

**Published:** 2025-07-28

**Authors:** Mei Zhang, Feng Yang

**Affiliations:** 1 School of Mathematics, Southwest Minzu University, Chengdu 610225, China; zhangmei150320@163.com; 2 School of Mathematical Sciences, Sichuan Normal University, Chengdu 610066, China

**Keywords:** federated semi-supervised learning, convergence rate, linear speedup, quasi-Monte Carlo techniques, partial client participation

## Abstract

Federated semi-supervised learning (Fed-SSL) has emerged as a powerful framework that leverages both labeled and unlabeled data distributed across clients. To reduce communication overhead, real-world deployments often adopt partial client participation, where only a subset of clients is selected in each round. However, under non-i.i.d. data distributions, the choice of client sampling strategy becomes critical, as it significantly affects training stability and final model performance. To address this challenge, we propose a novel federated averaging semi-supervised learning algorithm, called FedAvg-SSL, that considers two sampling approaches, uniform random sampling (standard Monte Carlo) and a structured lattice-based sampling, inspired by quasi-Monte Carlo (QMC) techniques, which ensures more balanced client participation through structured deterministic selection. On the client side, each selected participant alternates between updating the global model and refining the pseudo-label model using local data. We provide a rigorous convergence analysis, showing that FedAvg-SSL achieves a sublinear convergence rate with linear speedup. Extensive experiments not only validate our theoretical findings but also demonstrate the advantages of lattice-based sampling in federated learning, offering insights into the interplay among algorithm performance, client participation rates, local update steps, and sampling strategies.

## 1. Introduction

### 1.1. Background

With the exponential growth of decentralized data generated by devices such as smartphones, federated learning (FL) [1,2] provides a framework for training high-quality shared global models while preserving data privacy. However, compared with the decentralized optimization [3,4], FL gives rise to significant challenges, particularly in terms of communication efficiency and client availability. To address these challenges, various FL algorithms have been developed [2], with federated averaging (FedAvg) [1] being one of the most widely used. FedAvg works by randomly selecting a subset of clients, performing local stochastic gradient descent (SGD) [5] on the selected clients, and aggregating the resulting models at the server [6]. Numerous studies have analyzed the convergence of FedAvg [7] and have proposed its variations [8,9,10], particularly in scenarios with partial client participation.

Existing federated learning approaches [11,12] commonly assume a supervised learning setup, where local private data is fully labeled. However, data labeling is often expensive and time-consuming, while unlabeled data is readily available in abundance. This drives us to effectively leverage these massive, distributed pools of unlabeled data to enhance federated learning. A straightforward approach might be to apply semi-supervised learning (SSL) techniques [13,14,15] to handle unlabeled data, while using traditional federated learning algorithms to aggregate the learned weights.

### 1.2. Related Works

**Semi-Supervised Learning.** Numerous studies have explored centralized semi-supervised learning. One prominent approach is pseudo-labeling [16], which generates one-hot labels from highly confident predictions on unlabeled data and uses these labels as training targets with a standard cross-entropy loss. Another major category of SSL methods involves consistency regularization. These methods, such as the ladder network [17], the Π model [18], the mean teacher [19], virtual adversarial training (VAT) [20,21], and MixMatch [22], assume that the class semantics remain invariant under transformations of the input instances. To leverage this assumption, they enforce the model’s predictions to be consistent across different perturbations of the input data. Notably, Du et al. [23] further advanced this direction by proposing label propagation for imbalanced multi-label classification, and Ref. [24] developed specialized augmentation strategies for semi-supervised medical image segmentation.

**Federated Semi-Supervised Learning.** Federated semi-supervised learning (Fed-SSL) [25] extends traditional federated learning to scenarios characterized by limited labeled data and abundant distributed unlabeled data. Notable methods in this area include FedSem [26], which trains a global model on labeled data and incorporates unlabeled data using pseudo-labeling. Jeong et al. [27] introduced an inter-client consistency loss to align predictions across clients, enhancing the utilization of unlabeled data. Recently, Wang et al. [28] proposed a personalized Fed-SSL framework that leverages adaptive variance reduction and normalized aggregation to address client heterogeneity and improve convergence. Similarly, Liang et al. [29] developed RSCFed, which constructs multiple sub-consensus models by randomly sampling clients and then aggregates these sub-consensus models into the global model for improved reliability and performance. Ref. [30] proposed a two-stage sampling method that uses the predicted distribution changes of samples after different data augmentations.

**Partial Client Participation.** In federated learning (FL), clients may randomly join or leave the system, leading to a stochastic and time-varying set of active participants across communication rounds. For FL with non-i.i.d. datasets and partial client participation [31], several advancements have been made. Yang et al. [7] established a linear speedup in the convergence of FedAvg under partial client participation. Wang et al. [11] provided a unified convergence analysis for FL with arbitrary client participation, and Wang et al. [32] proposed FedAU, which adaptively weights client updates using online estimates of optimal weights without requiring prior knowledge of participation statistics. Ref. [33] considered a variance reduction algorithm applied at the server that eliminates error due to partial client participation.

Despite these efforts, most convergence analyses focus solely on traditional FL. In the context of federated semi-supervised learning, especially with non-i.i.d. datasets and partial client participation, the convergence behavior remains underexplored. A fundamental question arises: Can an algorithm in Fed-SSL still achieve the same linear speedup for convergence under non-i.i.d. data distributions and varying degrees of client participation? Addressing this challenge is crucial for extending the theoretical foundations of Fed-SSL and ensuring its robustness in practical applications.

**Quasi-Monte Carlo Methods in Subsampling.** The challenge of partial client participation in FL shares similarities with high-dimensional numerical integration. Quasi-Monte Carlo (QMC) methods [34], rooted in number-theoretic techniques [35], address this by constructing low-discrepancy sequences that achieve superior uniformity in sample distribution—akin to the uniform design in experimental design theory. Zhang et al. [36] and Zhou et al. [37] proposed an efficient model-free subsampling method based on uniform design, which generates representative subdata from the original dataset. This inspires us to similarly employ quasi-Monte Carlo approaches to address the partial client participation.

### 1.3. Contributions

In this paper, we tackle the federated semi-supervised learning problem with partial client participation, which introduces significant complexity compared to traditional federated optimization problems. Specifically, Fed-SSL involves two sets of optimization variables: the global model and the pseudo-labels, along with the added challenge of constraints. As a result, proving the convergence of algorithms for this problem is notably more difficult.

The main contributions of this paper are as follows:

- We propose an efficient federated learning algorithm, FedAvg-SSL, which incorporates partial client participation and alternates between updating the global model and refining pseudo-labels on local clients. The algorithm supports both uniform random sampling and a more structured lattice-based sampling strategy at the server side. While uniform sampling ensures simplicity and unbiased selection, the lattice-based approach offers more balanced client participation, which improves model stability under non-i.i.d. data.
- We establish a rigorous convergence analysis for FedAvg-SSL, demonstrating a sublinear convergence rate and linear speedup under partial client participation.
- Experimental results validate the performance of the proposed algorithm, showing its consistency with theoretical findings and illustrating the relationship between algorithm performance, the number of participating clients, and the number of local steps.

### 1.4. Organization

The rest of the paper is organized as follows. Section 2 introduces the federated SSL optimization problem. In Section 3, we present an efficient federated SSL algorithm that incorporates partial client participation via both uniform random sampling and a more structured lattice-based sampling strategy at the server side. In addition, the proposed algorithm alternates between updating the global model and refining pseudo-labels on local clients.

Section 4 provides a convergence rate analysis of the proposed algorithm. In Section 5, we discuss the practical implications of our results. Finally, we conclude in Section 6, with all proofs deferred to the appendix.

## 2. Problem Formulation

Consider a FL setting with a server and *K* distributed clients. We assume that both labeled data L and unlabeled data U are distributed across these *K* clients. Specifically, for each client *k*, the local dataset $D_{k}$ consists of both labeled and unlabeled data, i.e.,

$$D_{k}=L_{k}\cup U_{k},$$

where $L_{k}=\left\{\left(x_{k,i},y_{k,i}\right),i=1,\ldots ,N_{k}\right\}$ is the local labeled dataset and $U_{k}=\left\{u_{k,i},i=1,\ldots ,M_{k}\right\}$ is the local unlabeled dataset. Here, $x_{k,i}$ is the *i*-th labeled sample for the *k*-th client with the corresponding label $y_{k,i}\in R^{C}$, which is a one-hot vector representing the true class label, and *C* is the number of classes. Similarly, $u_{k,i}$ is the *i*-th unlabeled sample for the *k*-th client. The datasets $D_{k}$, for k∈[K]≜{1,…,K}, are assumed to be non-overlapping. Additionally, $N_{k}$ and $M_{k}$ denote the number of labeled and unlabeled samples, respectively, for client *k*.

Let $\hat{y}=\left\{{\hat{y}}_{1},\ldots ,{\hat{y}}_{K}\right\}$ denote the collection of pseudo-labels across all clients, where ${\hat{y}}_{k}=\left\{{\hat{y}}_{k,1},\ldots ,{\hat{y}}_{k,M_{k}}\right\}$ represents the set of pseudo-labels for the *k*-th client. Each pseudo-label ${\hat{y}}_{k,i}\in R^{C}$ belongs to the feasible set $Y_{k}=\left\{{\hat{y}}_{k}\;|\;e^{⊤}{\hat{y}}_{k,i}=1,\;{\hat{y}}_{k,i}\geq 0,i\in \left[M_{k}\right]\right\}$, ensuring proper probability distributions. Following [38], we formulate the Fed-SSL optimization problem as follows:

(1a) $$\begin{array}{cc} \min_{\begin{array}{c} \theta ,\hat{y} \end{array}}\; & F\left(\theta ,\hat{y}\right)≜\frac{1}{K}\sum_{k=1}^{K}\left[\underset{≜F_{k}\left(\theta ,{\hat{y}}_{k}\right)}{\underset{︸}{\ell_{k}\left(\theta ,{\hat{y}}_{k}\right)+\alpha_{1}r_{1}\left({\hat{y}}_{k}\right)}}\right] \end{array}$$

(1b) $$\begin{array}{cc} s.t.\; & {\hat{y}}_{k}\in Y_{k},\;k\in \left[K\right]. \end{array}$$

The loss function for each client consists of two components: a supervised loss on labeled data and a pseudo-labeled loss on unlabeled data, defined as follows: $\ell_{k}\left(\theta ,{\hat{y}}_{k}\right)=L_{\mathrm{CE}}\left(\theta ;L_{k}\right)+\alpha_{0}L_{\mathrm{CE}}\left(\theta ;U_{k},{\hat{y}}_{k}\right)$ where the cross-entropy loss is given by the following:

$$\begin{array}{c} L_{\mathrm{CE}}\left(\theta ;L\right)=-\frac{1}{N}\sum_{i=1}^{N}\langle y_{i},\log\left(f_{\theta }\left(x_{i}\right)\right)\rangle . \end{array}$$

To prevent overconfident pseudo-labels, we introduce a regularization term $r_{1}\left({\hat{y}}_{k}\right)$:

(2) $$\begin{array}{c} r_{1}\left({\hat{y}}_{k}\right)=\frac{1}{M_{k}}\sum_{i=1}^{M_{k}}\mathrm{KL}\left({\hat{y}}_{k,i},u\right), \end{array}$$

where $u=\left[\frac{1}{C},\ldots ,\frac{1}{C}\right]\in R^{C}$ represents a uniform distribution, and KL(·,·) denotes the Kullback–Leibler divergence.

The optimization problem in (1) presents two main challenges: (a) it involves two groups of variables (θ and $\hat{y}$), and (b) the pseudo-labels must satisfy probability simplex constraints. These characteristics naturally suggest an alternating optimization approach for updating these variables.

## 3. FedAvg-SSL Algorithm

In this section, we propose the FedAvg semi-supervised learning (FedAvg-SSL) algorithm as a solution approach to the optimization problem formulated in (1).

The FedAvg-SSL framework operates under the standard federated learning paradigm, where a central server coordinates training across *K* distributed clients. In each communication round *t*, the server selects a subset of clients $S^{t}\subseteq K=\left\{1,2,\ldots ,K\right\}$ of fixed size *M* to participate in model updates. When M=K, all clients are involved in every round. In this section, we explore two distinct strategies for determining the active client subset $S^{t}$ at each round *t*.

### 3.1. Random Sampling Method

The random sampling method in federated learning selects clients for each round independently and uniformly from the entire client. Let K={1,2,…,K} be the set of all clients. For each iteration t∈{0,1,…,T−1}, we select a subset of clients $S^{t}$ via uniform random sampling without replacement from set K with size *M*; then, the probability of any client being selected in a round is

$$\begin{array}{c} P\left(i\in S^{t}\right)=\frac{M}{K},\forall \;i=1,\ldots ,K. \end{array}$$

Although each client has the same probability of being selected in each round, the random sampling method can lead to uneven client participation where some clients might be selected multiple times while others might never be selected, especially when the number of communication rounds is limited.

### 3.2. Lattice-Based Sampling Method

To improve participation uniformity, we propose a lattice-based sampling method that leverages a uniform design to construct a deterministic selection matrix. This method aims to ensure that all clients are sampled more evenly across communication rounds.

Let *T* be the number of total rounds and *M* be the number of clients selected per round. Typically, the selected subset size *M* is a divisor of the total client number *K*. Let q=K/M, then the set of all clients K={1,2,…,K} could be divided into *M* groups with *q* clients in each group: {1,2,…,q},{q+1,q+2,…,2q},…,{(M−1)q+1,(M−1)q+2,…,K}. Our selection strategy requires choosing exactly one client from each of *M* predefined groups, resulting in *M* selected clients per round. It is noticed that each group contains *q* distinct clients, which naturally evokes the concept of *q*-level uniform designs in experimental design theory. The uniform designs always have a good space-filling property on the experimental domain. We treat the *M* groups, each containing *q* distinct clients, as the value ranges for *M* factors in the experimental design framework. To ensure that all clients are sampled more evenly across *T* rounds, we perform sampling according to the rows of the uniform design.

Let $U=U_{T}\left(q^{M}\right)$ denote a uniform design comprising *T* runs for *M* factors, each having *q* levels, such that we

(1) Let U(t,k)∈{1,2,…,q} denote the element in the *t*-th row and *k*-th column of the matrix *U* for all t=0,…,T−1,k=1,…,K;
(2) Each level appears equally often in every column.

To obtain a uniform design $U=U_{T}\left(q^{M}\right)$, the first method is finding designs straightforwardly from the library of uniform designs given by [39], which does not require any calculations. If there is no such design in the library, two other methods can be used to construct such a design. Let k(T)=ϕ(T)/2+1, where ϕ(·) is the Euler function. If M<k(T+1), the leave-one-out good lattice point method combined with pseudo-level transformations [40] can be used to construct a nearly uniform design. For the other cases, we can use the R package UniDOE (proposed by [41]) on R version 3.4.1 to search for a nearly uniform design. Actually, the UniDOE package can be used to search for a nearly uniform design for arbitrary T,M. However, the UniDOE package is a little slower than the leave-one-out good lattice point method in general; then, we recommend to use the former only when M≥k(T+1).

Based on the uniform design *U*, the sampling design *D* is obtained by a deterministic offset

(3) $$\begin{array}{c} D(t,k)=U(t,k)+(k-1)q,\;\forall \;t=0,\ldots ,T-1,\;\forall \;k=1,\ldots ,K. \end{array}$$

Then, all elements of the sampling design *D* are the actual client IDs. At each communication round *t*, one row of the matrix *D* is selected (either sequentially or randomly without replacement), and the resulting client indices form the active set $S^{t}$.

**Figure 1:**
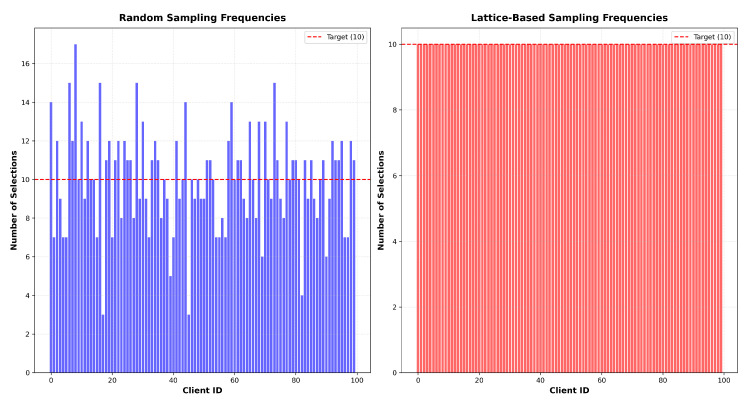
A toy example of our client selection procedure where we sequentially select 10 clients from 100 clients in total over 100 communication rounds. The red dashed line indicates the mean selection count (10) in the non-sampling scheme. The left subplot shows the random sampling pattern where some clients might be selected multiple times (blue bars) while others might be rarely selected, while the right subplot demonstrates our lattice-based sampling method that ensures each client is selected exactly 10 times (red bars) with a systematic and balanced distribution.

This lattice-based scheme systematically spreads participation over time, significantly reducing the risk of client over-sampling or under-sampling (see Figure 1). As a result, it is particularly advantageous in federated semi-supervised learning scenarios, where balanced exposure to heterogeneous client data is crucial for effective model generalization.

### 3.3. The Proposed Federated SSL Algorithm

In this subsection, we present the proposed Fed-SHVR algorithm for solving problem (1), and its detailed pseudo code is presented in Algorithms 1 and 2. Since the considered problem (1) involves two blocks of variables θ and $\hat{y}$, we propose to train a global model for (1) in a novel way that combines an alternatively updating strategy with the local SGD algorithm.

Specifically, for each communication round, our algorithm has two parts: one is the client update and the other is the server update.

**Client Side.** After the server broadcasts the current global model $\theta^{t}$ to all active clients in $S^{t}$, each active client *k* first updates its pseudo-labels by solving the following optimization problem:

(4a) $$\begin{array}{cc} {\hat{y}}_{k}^{t+1}={\arg\min}_{\begin{array}{c} {\hat{y}}_{k} \end{array}}\; & \alpha_{0}L_{\mathrm{CE}}\left(\theta^{t};u_{k},{\hat{y}}_{k}\right)+\alpha_{1}r_{1}\left({\hat{y}}_{k}\right) \end{array}$$

(4b) $$\begin{array}{cc} s.t.\; & {\hat{y}}_{k}\in Y_{k}. \end{array}$$

Following [38], this optimization problem admits a closed-form solution:  

(5) $$\begin{array}{c} {\left[{\hat{y}}_{k,i}^{t+1}\right]}_{j}=\frac{{\left[f_{\theta^{t}}\left(u_{k,i}\right)\right]}_{j}^{\frac{\alpha_{0}}{\alpha_{1}}}}{\sum_{j=1}^{C}{\left[f_{\theta^{t}}\left(u_{k,i}\right)\right]}_{j}^{\frac{\alpha_{0}}{\alpha_{1}}}},j=1,\ldots ,C, \end{array}$$

where ${\left[{\hat{y}}_{k,i}\right]}_{j}$ denotes the *j*-th entry of ${\hat{y}}_{k,i}$. After updating the pseudo-labels, each client performs local model updates:

- Samples mini-batches of labeled data $\xi_{k}$ and unlabeled data $\zeta_{k}$ uniformly at random from $L_{k}$ and $U_{k}$, respectively.
- Computes the stochastic gradient of the local loss function
  (6) $$\begin{array}{c} g_{k}\left(\theta_{k},{\hat{y}}_{k}\right):=\nabla_{\theta }F_{k}\left(\theta_{k},{\hat{y}}_{k};\xi_{k},\zeta_{k}\right). \end{array}$$
- Updates its local model copy $\theta_{k}$ using SGD.

**Server Side.** Each active client transmits its updated local model $\theta_{k,Q}$ back to the server, which aggregates these updates to compute the new global model $\theta^{t+1}$ via (7).
**Algorithm 1** FedAvg SSL                                                       [Server]1:**Input:** initial model parameters $\theta^{0}$.2:**for** communication round t=0 **to** T−1 **do**3:    Sample clients $S^{t}$ uniformly randomly so that $|S^{t}|=M$, or according to the sampling design *D* obtained by (3).4:    **for** each client $k\in S^{t}$ **in parallel do**5:      Send $\theta^{t}$ to client *k*.6:      Receive $\theta_{k}^{t+1}$ from client *k* via Algorithm 2.7:    **end for**8:    Update global model(7)$$\begin{array}{c} \theta^{t+1}=\left(1-\eta_{\theta }\right)\theta^{t}+\frac{\eta_{\theta }}{M}\sum_{k\in S^{t}}\theta_{k}^{t+1}. \end{array}$$9:**end for**

**Algorithm 2** FedAvg SSL                       [Client]
1:Receive $\theta^{t}$ from the server.2:Update pseudo-label ${\hat{y}}_{k}^{t+1}$ from (5).3:Initialize $\theta_{k,0}^{t}=\theta^{t}$.4:
**for** q=0,…,Q−1 **do**
5:    Select data $\xi_{k,q}^{t}$ and $\zeta_{k,q}^{t}$ uniformly at random from $L_{k}$ and $U_{k}$.6:    Update local client model(8)$$\begin{array}{c} \theta_{k,q+1}^{t}=\theta_{k,q}^{t}-\eta_{g}g_{k}\left(\theta_{k,q}^{t},{\hat{y}}_{k}^{t+1}\right). \end{array}$$7:
**end for**
8:Set $\theta_{k}^{t+1}=\theta_{k,Q}^{t}$.9:Send $\theta_{k}^{t+1}$ back to the server.


## 4. Convergence Analysis with Partial Client Participation

In this section, we establish the convergence properties of FedAvg-SSL when only a subset of clients updates their variables per outer loop.

### 4.1. Assumptions

We first make some standard assumptions.

**Assumption** **1.** *The regularization term $r_{1}\left(\hat{y}\right)$ is a continuously differentiable function. Furthermore, $r_{1}\left(\hat{y}\right)$ is μ-strongly convex, where μ>0. Specifically, for any ${\hat{y}}_{1},{\hat{y}}_{2}\in Y_{k}$, the following inequality holds:*

(9) $$\begin{array}{c} r_{1}\left({\hat{y}}_{1}\right)-r_{1}\left({\hat{y}}_{2}\right)-\langle \nabla r_{1}\left({\hat{y}}_{2}\right),{\hat{y}}_{1}-{\hat{y}}_{2}\rangle \geq \frac{\mu }{2}{∥{\hat{y}}_{1}-{\hat{y}}_{2}∥}^{2}. \end{array}$$

The regularization term $r_{1}\left({\hat{y}}_{k}\right)$ defined in (2) is strongly convex over the probabilistic simplex $Y_{k}$ (see [42], Definition 2 and Example 2). However, it is important to note that the objective function $F_{k}\left(\theta ,{\hat{y}}_{k}\right)$ in (1a) is not jointly convex with respect to $\left(\theta ,\hat{y}\right)$.

**Assumption** **2.** *The local cost $F_{k}\left(\theta ,{\hat{y}}_{k}\right)$ is L-smooth (possibly non-convex) with respect to $\left(\theta ,{\hat{y}}_{k}\right)$ for k∈[K], i.e.,*

(10) $$\begin{array}{c} ∥\nabla_{\theta }F_{k}\left(\theta ,{\hat{y}}_{k}\right)-\nabla_{\theta }F_{k}\left(\theta^{\prime },{\hat{y}}_{k}^{\prime }\right)∥\leq L\sqrt{∥\theta -\theta^{\prime }{∥}^{2}+{∥{\hat{y}}_{k}-{\hat{y}}_{k}^{\prime }∥}^{2}}, \end{array}$$

*for all $\theta ,\theta^{\prime }$ and ${\hat{y}}_{k},{\hat{y}}_{k}^{\prime }\in Y_{k}$.*

**Assumption** **3.** *Given ${\hat{y}}_{k}$, for any k∈[K], the stochastic gradient is unbiased for any θ. Specifically, the following conditions hold:*

(11) $$\begin{array}{cc} \; & E\left[g_{k}\left(\theta_{k},{\hat{y}}_{k}\right)\right]=\nabla_{\theta }F_{k}\left(\theta_{k},{\hat{y}}_{k}\right), \end{array}$$

(12) $$\begin{array}{cc} \; & E[∥g_{k}\left(\theta_{k},{\hat{y}}_{k}\right)-\nabla_{\theta }F_{k}\left(\theta_{k},{\hat{y}}_{k}\right){∥}^{2}]\leq \sigma_{\theta }^{2}, \end{array}$$

*where $\sigma_{\theta }$ represents the noise variance, and E denotes the expectation with respect to all random variables $\left\{\xi_{k},\zeta_{k}\right\}$.*

**Assumption** **4** (Bounded Gradient Dissimilarity). *There exists a constant $\sigma_{G}>0$ such that for any θ and $\hat{y}$, the following inequality holds:*

(13) $$\begin{array}{c} \frac{1}{K}\sum_{k=1}^{K}{∥\nabla_{\theta }F_{k}\left(\theta ,{\hat{y}}_{k}\right)-\nabla_{\theta }F\left(\theta ,\hat{y}\right)∥}^{2}\leq \sigma_{G}^{2}. \end{array}$$

Assumptions 2 and 3 are standard in first-order stochastic algorithms, while Assumption 4 constrains the data heterogeneity by bounding the gradient dissimilarity between different clients, which is commonly used in [7,11,28].

### 4.2. Convergence Analysis of FedAvg-SSL

From (7) and (8), the update for the global model across consecutive outer loops is given by

(14) $$\begin{array}{cc} \theta^{t+1}-\theta^{t} & =\frac{\eta_{\theta }}{M}\sum_{k\in S^{t}}\left(\theta_{k}^{t+1}-\theta^{t}\right)=-\frac{\eta }{M}\sum_{k,q}I_{k\in S^{t}}g_{k}\left(\theta_{k,q}^{t},{\hat{y}}_{k}^{t+1}\right), \end{array}$$

where $\eta =\eta_{\theta }\gamma_{\theta }$ and $I_{k\in S^{t}}$ denote the characteristic function of the set $S^{t}$, representing the subset of clients participating in the current round.

A critical challenge arises because the right-hand side of the above equation does not provide an unbiased estimation of the full gradient. Specifically, we have

(15) $$\begin{array}{c} \frac{1}{M}\sum_{k,q}E\left[I_{k\in S^{t}}g_{k}\left(\theta_{k,q}^{t},{\hat{y}}_{k}^{t+1}\right)\right]\neq \nabla F\left(\theta ,y^{t+1}\right), \end{array}$$

where $\nabla F(\theta ,y^{t+1})$ is the gradient of the full objective function.

This discrepancy stems from the stochastic nature of partial client participation, as only a subset of clients $S^{t}$ is actively involved in each communication round. Consequently, the aggregated gradient $\frac{1}{M}\sum_{k,q}I_{k\in S^{t}}g_{k}$ introduces bias, which complicates the convergence analysis. Specifically, controlling this bias becomes essential to establish rigorous convergence guarantees for FedAvg-SSL under partial client participation.

Before we show the convergence, let us first define the following optimality gap:

(16) $$\begin{array}{c} g_{t}≜E[∥\nabla_{\theta }F\left(\theta^{t},{\hat{y}}^{t+1}\right){∥}^{2}]+\frac{1}{K}\sum_{k=1}^{K}E[∥{\hat{y}}_{k}^{t+1}-{\hat{y}}_{k}^{t}{∥}^{2}]. \end{array}$$

which measures how far an iteration point is away from the stationary point. From the definition, we know $g_{t}\geq 0$. If $g_{t}=0$, the iterate $\left\{\theta^{t},{\hat{y}}^{t}\right\}$ will be a stationary point of problem (1). According to (16), we have

(17) $$\begin{array}{c} \nabla_{\theta }F\left(\theta^{t},{\hat{y}}^{t}\right)=0,\;{\hat{y}}_{k}^{t+1}-{\hat{y}}_{k}^{t}=0. \end{array}$$

Since ${\hat{y}}_{k}^{t+1}$ is the optimal solution of $F_{k}\left(\theta^{t},\hat{y}\right)$, from the first order optimality condition, we get

(18) $$\begin{array}{c} \langle \nabla_{{\hat{y}}_{k}}F_{k}\left(\theta^{t},{\hat{y}}_{k}^{t+1}\right),{\hat{y}}_{k}-{\hat{y}}_{k}^{t+1}\rangle \geq 0,\;\mathrm{for}\;\forall \;{\hat{y}}_{k}\in Y_{k}. \end{array}$$

Combing (17) and (18), for $\forall \;\theta ,{\hat{y}}_{k}\in Y_{k}$, we have

$$\begin{array}{c} \langle \nabla_{\theta }F\left(\theta^{t},{\hat{y}}^{t}\right),\theta -\theta^{t}\rangle +\sum_{k=1}^{K}\langle \nabla_{{\hat{y}}_{k}}F_{k}\left(\theta^{t},{\hat{y}}_{k}^{t}\right),{\hat{y}}_{k}-{\hat{y}}_{k}^{t}\rangle \geq 0. \end{array}$$

The above inequality implies that $\left\{\theta^{t},{\hat{y}}^{t}\right\}$ is a stationary point to problem (1).

**Theorem** **1.** *If $\eta_{g}^{2}\leq \frac{1}{64Q\left(Q-1\right)L^{2}}$ and $\eta :=\eta_{g}\eta_{\theta }\leq \frac{M(K-1)}{\left(3+12Q)QL(K-M\right)}$, we have that the sequence $\left\{\theta^{t},{\hat{y}}^{t}\right\}$ generated by Algorithm 1 satisfies*

(19) $$\begin{array}{cc} \frac{1}{T}\sum_{t=0}^{T-1}g_{t} & \leq \frac{4E[F\left(\theta^{0},{\hat{y}}^{0}\right)]}{\eta QT}+\eta_{g}^{2}B_{1}+4\eta \eta_{g}^{2}B_{2}+4\eta B_{3}, \end{array}$$

*where*

(20) $$\begin{array}{cc} B_{1} & =L^{2}\left(Q\left(Q-1\right)\right)\sigma_{G}^{2}+2L^{2}\left(Q-1\right)\sigma_{\theta }^{2}, \end{array}$$

(21) $$\begin{array}{cc} B_{2} & =\frac{24Q^{3}L^{3}\left(K-M\right)}{M(K-1)}\sigma_{G}^{2}+\frac{6Q\left(Q-1\right)L^{3}\left(K-M\right)}{M(K-1)}\sigma_{\theta }^{2}, \end{array}$$

(22) $$\begin{array}{cc} B_{3} & =\frac{3LQ(K-M)}{2M(K-1)}\sigma_{G}^{2}+\frac{L}{2M}\sigma_{\theta }^{2}. \end{array}$$

**Proof.**  See Appendix A. □

**Remark** **1.** *Theorem 1 provides an explicit upper bound on the optimality gap, which offers useful insights into how several key factors influence the convergence behavior of the proposed FedAvg-SSL algorithm:*

- *Number of participating clients (M): When all clients participate in each round, i.e., M=K, the term $B_{2}$ in the bound becomes zero. As a result, the convergence rate improves, demonstrating the benefit of increased client participation.*
- *Number of local steps (Q): As Q increases, the accumulated local update noise can affect convergence. Theorem 1 suggests that in such cases, the global and local learning rates η and $\eta_{g}$ should be chosen smaller to maintain stability and ensure convergence.*

*These observations provide practical guidance for selecting different parameters in FedAvg-SSL.*

With appropriate step sizes, we can derive the following corollary.

**Corollary** **1.** *Let $\eta_{g}=\frac{1}{\sqrt{T}QL}$ and $\eta =\frac{\sqrt{M}}{\sqrt{TQ}L}$; the convergence rate of the FedAvg-SSL algorithm under partial client participation is given by*

(23) $$\begin{array}{cc} \min_{t=0,\ldots ,T-1}g_{t} & \leq O\left(\frac{1}{\sqrt{MTQ}}+\frac{1}{T}\right). \end{array}$$

**Remark** **2.** *Corollary 1 demonstrates that the required number of outer loop iterations T to achieve an ϵ-solution decreases linearly as M increases. This phenomenon is commonly referred to as linear speedup with respect to M. To the best of our knowledge, this is the first work to establish the linear speedup of a federated semi-supervised learning algorithm.*

## 5. Simulation Results

We conducted extensive experiments on the MNIST dataset to evaluate the effectiveness of our proposed federated semi-supervised learning approach. The experiments were implemented using PyTorch version 1.8 on a CUDA-enabled GPU, utilizing the MNIST dataset which contains 60,000 training images and 10,000 test images of handwritten digits.

To simulate a realistic federated learning scenario, we configured our system with 100 clients (K=100) and implemented a partial participation strategy where only 10% of clients (10 clients) participate in each federation round. The dataset was divided into labeled and unlabeled portions, with 30% of the data being labeled and the remaining 70% unlabeled. We investigated two distinct data distribution scenarios:

- IID Setting: The training data is randomly and uniformly distributed across clients, ensuring each client receives approximately equal amounts of both labeled and unlabeled samples. This setting serves as our baseline distribution.
- Non-IID Setting: Labeled data is randomly shuffled and split into twice as many shards as there are clients, with each client randomly assigned two unique shards. This ensures that each client receives a distinct, non-overlapping subset of the labeled data, with each shard containing a random mix of classes. The unlabeled data is distributed using a Dirichlet distribution (α=0.01) to further enhance the non-IID nature of the data across clients.

For our model architecture, we employed a simple multi-layer perceptron (MLP) with one hidden layer containing 256 units and ReLU activations. The training process utilized a mini-batch size of 32 for both labeled and unlabeled data, with learning rates set to $\eta_{g}=\eta_{\theta }=0.01$. Each participating client performed 10 local epochs during their training phase.

**Impact of hyperparameters $\alpha_{0}$ and $\alpha_{1}$.** The hyperparameter $\alpha_{0}$ controls the model’s reliance on the unlabeled data during training. To prevent the model from prematurely overfitting to noisy pseudo-labels, we adopt a gradually increasing schedule for $\alpha_{0}$, inspired by the work in [16]. Specifically, we define

$$\alpha_{0}=\left\{\begin{array}{cc} \exp\left(-5{\left(1-\frac{t}{T_{1}}\right)}^{2}\right), & \mathrm{if}\;t<T_{1}\\ 1, & \mathrm{if}\;t\geq T_{1}. \end{array}\right.$$

The second hyperparameter, $\alpha_{1}$, serves as a regularization weight in the pseudo-label refinement process. It controls the entropy of the pseudo-labels: smaller values encourage confident (i.e., lower-entropy) predictions, while larger values encourage more conservative updates by penalizing overconfidence. Thus, tuning $\alpha_{1}$ balances the trade-off between exploration and exploitation of pseudo-labels.

**Figure 2:**
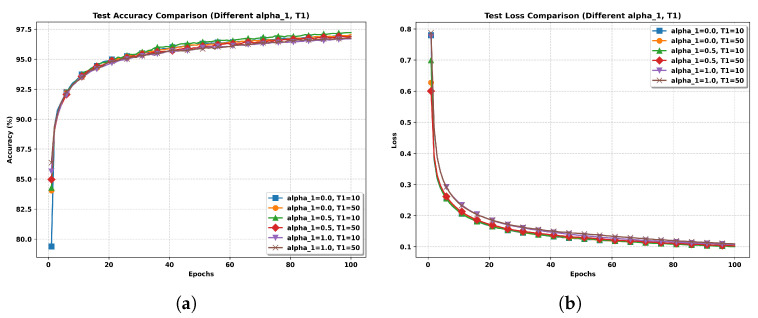
Performance comparison of FedAvg-SSL approaches under different parameter settings. (**a**) Test Accuracy Comparison. (**b**) Test Loss Comparison.

To investigate the impact of these hyperparameters, we conduct a sensitivity analysis and summarize the results in the table below. The test accuracy is evaluated under different combinations of $T_{1}$ and $\alpha_{1}$ in the non-IID setting. As shown in Table 1, the highest accuracy of 97.5% occurs when $T_{1}=10$ and $\alpha_{1}=0.5$, outperforming other configurations. Based on these results, we adopt this parameter configuration for all subsequent experiments. Figure 2 further illustrates the effects of these parameters on training dynamics.

**Figure 3:**
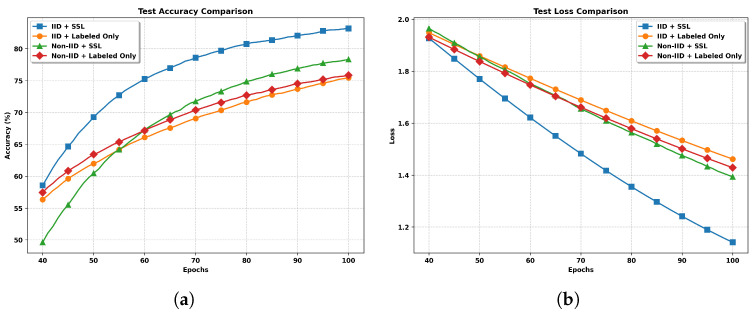
Performance comparison of federated learning approaches under different data distribution settings. The results show the superiority of SSL methods over supervised-only approaches in both IID and non-IID scenarios. (**a**) Test Accuracy Comparison. (**b**) Test Loss Comparison.

**Impact of non-IID datasets.** As illustrated in Figure 3a, we present comprehensive experimental results comparing the performance of federated semi-supervised learning under both IID and non-IID data distributions. The test accuracy curves in Figure 3a demonstrates that the non-IID data distribution significantly impacts the model’s convergence and final performance. Under the IID setting with SSL, the model achieves the highest accuracy of approximately 83%, while the same approach under non-IID conditions reaches only 78%, indicating a clear performance degradation due to data heterogeneity.

A key observation from the loss curves in Figure 3b is that the non-IID data distribution notably slows down the convergence rate. The IID+SSL configuration shows the fastest convergence and reaches the lowest loss value of approximately 1.15, while its non-IID counterpart maintains a consistently higher loss of around 1.4. This pattern aligns with theoretical expectations that higher degrees of data heterogeneity lead to slower convergence and higher final loss values.

Furthermore, the impact of non-IID data is evident in both SSL and supervised-only approaches, though with varying degrees. The performance gap between IID and non-IID settings is more pronounced in the SSL approach (5% difference) compared to the supervised-only setting (1% difference). This observation suggests that while SSL techniques can effectively leverage unlabeled data, their benefits are somewhat diminished under non-IID conditions, highlighting the fundamental challenges that data heterogeneity poses in federated learning scenarios.

**Figure 4:**
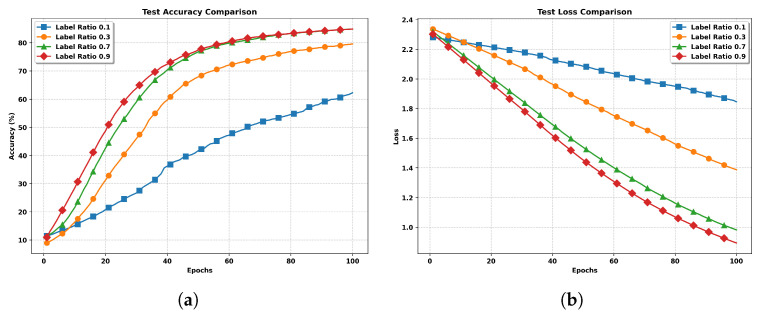
Performance comparison with different label ratios (0.1, 0.3, 0.7, and 0.9) under non-IID settings. Higher label ratios generally lead to better performance and faster convergence. (**a**) Test Accuracy with Different Label Ratios. (**b**) Test Loss with Different Label Ratios.

**Impact of label ratios.** To investigate how the proportion of labeled data affects model performance, we conducted experiments with varying label ratios. Figure 4 presents the test accuracy and loss curves for different label ratios ranging from 0.1 to 0.9.

Figure 4a demonstrates a clear correlation between label ratio and model performance. With a high label ratio of 0.9, the model achieves the best performance, reaching approximately an 85% accuracy after 100 epochs. The convergence is also notably faster, showing steep improvement in the early stages (20–40 epochs). In contrast, with only 10% labeled data (ratio 0.1), the model struggles significantly, achieving only about 62% accuracy and showing much slower convergence throughout the training process. The loss curves in Figure 4b further reinforce these findings. Models trained with higher label ratios (0.7 and 0.9) demonstrate more efficient optimization, reaching lower loss values (around 0.9–1.0) and showing steeper descent curves.

**Figure 5:**
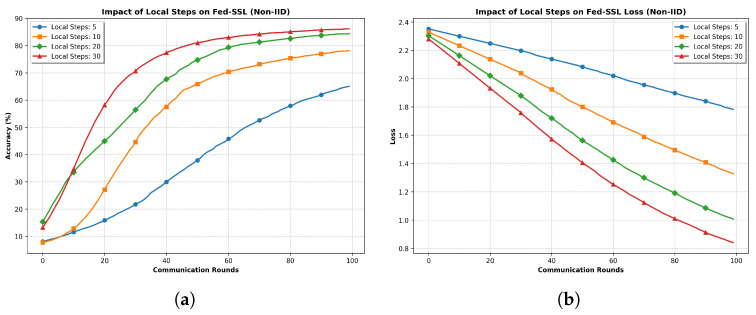
Impact of local training steps on Fed-SSL performance under non-IID settings. Increasing local steps leads to faster convergence and better final performance, consistent with Theorem 1. (**a**) Test Accuracy with Different Local Steps. (**b**) Test Loss with Different Local Steps.

**Impact of Local Training Steps.** To validate Theorem 1 and investigate the effect of local training steps on Fed-SSL’s performance under non-IID settings, we conducted experiments with varying numbers of local steps (5, 10, 20, and 30). Figure 5 presents the experimental results.

The experimental results strongly align with our theoretical analysis in Theorem 1 regarding the convergence of Fed-SSL. As shown in Figure 5a, increasing the number of local steps significantly improves both convergence speed and final model performance. With 30 local steps, the model achieves the highest accuracy of approximately 85% and demonstrates the fastest convergence rate, particularly during the early stages (20–40 communication rounds). In contrast, with only five local steps, the model shows slower convergence and reaches a lower final accuracy of about 73%.

The loss curves in Figure 5b further validate our theoretical findings. Models trained with more local steps (20 and 30) exhibit more efficient optimization trajectories, achieving lower final loss values (around 0.9–1.0) and showing steeper descent curves. This observation is consistent with Theorem 1, which suggests that increasing local training steps can enhance convergence stability and final performance, provided the learning rate is properly chosen.

**Figure 6:**
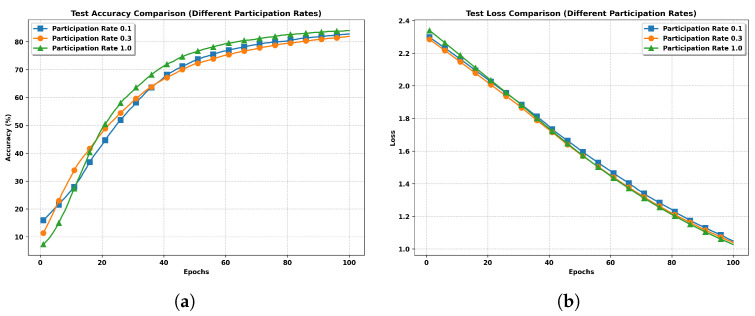
Performance comparison of FedSSL with different participation rates under non-IID setting. (**a**) Test Accuracy with Different Numbers of Clients. (**b**) Test Loss with Different Numbers of Clients.

**Impact of Client Number.** As shown in Figure 6, we compare the test accuracy and loss curves among three different participation rates: full worker participation (K=100) and partial worker participation (M=30 and M=10) under the same hyperparameter settings. The results demonstrate that full worker participation consistently outperforms partial participation scenarios, achieving approximately 83% accuracy compared to 82.5% and 82% for 10% and 30% participation rates, respectively. This performance gap can be attributed to the additional randomness introduced by partial worker participation, resulting in slower convergence rates as evidenced by the more gradual slopes of both accuracy and loss curves.

This phenomenon is particularly pronounced in our non-IID setting, where system heterogeneity plays a crucial role. Full worker participation effectively neutralizes this heterogeneity in each communication round by incorporating updates from all clients. In contrast, partial participation struggles to maintain this balance, as the selected subset of workers may not adequately represent the overall data distribution. For instance, with only 10 or 30 active workers, the available training data might cover only a subset of the MNIST dataset’s 10 digit classes, leading to biased model updates. This effect is clearly visible in both the accuracy and loss trajectories, where partial participation schemes exhibit consistently inferior performance throughout the training process.

**Figure 7:**
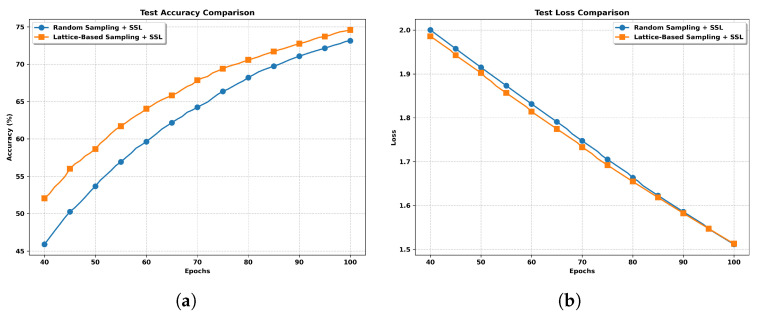
Performance comparison of FedSSL with different sampling strategies under non-IID setting. (**a**) Test Accuracy with Different Sampling Strategies. (**b**) Test Loss with Different Sampling Strategies.

**Impact of Sampling Strategies.** As shown in Figure 7, the lattice-based sampling consistently outperforms random sampling in both test accuracy and test loss. The reason may be that the lattice-based sampling method ensures that all clients contribute equally across communication rounds, effectively mitigating the bias introduced by data heterogeneity. In contrast, random sampling may result in some clients being over- or under-represented, leading to less representative model updates (see Figure 1).

## 6. Conclusions

In this paper, we propose an efficient FedAvg-SSL algorithm that alternately updates model parameters and pseudo-labels in non-IID federated settings for general non-convex optimization. To address the challenge of partial client participation, we introduce and compare two sampling strategies—uniform random sampling and lattice-based sampling—at the server side. The lattice-based method is shown to enhance participation balance and reduce selection variance across communication rounds. We provide a rigorous theoretical analysis demonstrating that FedAvg-SSL achieves a sublinear convergence rate with linear speedup under partial participation. Extensive experiments on the MNIST dataset support our theoretical claims and offer practical insights into how sampling strategy, participation rate, and local update steps jointly influence learning performance. These results provide valuable guidance for the design of efficient and robust federated semi-supervised learning systems. In future work, we plan to explore fairness-aware QMC-based client selection strategies, building on insights from [43], to ensure more equitable participation and performance across clients.

## Figures and Tables

**Table 1:** Test accuracy of the proposed algorithm by using different parameters $\alpha_{1}$ and $\alpha_{2}$ on the non-IID case.

	$T_{1}=10$	$T_{1}=50$
$\alpha_{1}=0$	96.91%	97.21%
$\alpha_{1}=0.5$	**97.5%**	96.89%
$\alpha_{1}=1$	96.71%	96.76%

## Data Availability

The data presented in this study are available on request from the first author.

## Appendix A. Proof of Theorem 1

For ease of presentation, we first show some important lemmas about the descent of the objective.

### Appendix A.1. Some Auxiliary Lemmas

The following lemma bounds the change in function value across consecutive outer loops for FedAvg-SSL with partial client participation.

**Lemma** **A1.**
*Under Assumptions 1 and 2, it holds that*

(A1)
$$\begin{array}{cc} E\left[F\left(\theta^{t+1},{\hat{y}}^{t+1}\right)\right]-E\left[F\left(\theta^{t},{\hat{y}}^{t}\right)\right]\leq & E\left[\langle \nabla_{\theta }F\left(\theta^{t},{\hat{y}}^{t+1}\right),\theta^{t+1}-\theta^{t}\rangle \right]+\frac{L}{2}E[∥\theta^{t+1}-\theta^{t}{∥}^{2}]\\ \; & -\frac{\mu \alpha_{1}}{2K}\sum_{k=1}^{K}E[∥{\hat{y}}_{k}^{t}-{\hat{y}}_{k}^{t+1}{∥}^{2}]. \end{array}$$

**Proof.** Firstly, we can establish the following equality:

(A2) $$\begin{array}{c} F_{k}\left(\theta^{t+1},{\hat{y}}_{k}^{t+1}\right)-F_{k}\left(\theta^{t},{\hat{y}}_{k}^{t}\right)=F_{k}\left(\theta^{t+1},{\hat{y}}_{k}^{t+1}\right)-F_{k}\left(\theta^{t},{\hat{y}}_{k}^{t+1}\right)+F_{k}\left(\theta^{t},{\hat{y}}_{k}^{t+1}\right)-F_{k}\left(\theta^{t},{\hat{y}}_{k}^{t}\right). \end{array}$$

Since each client $F_{k}$ has Lipschitz continuous gradients with respect to $\left(\theta ,{\hat{y}}_{k}\right)$ by Assumption 2, we have

(A3) $$\begin{array}{c} F_{k}\left(\theta^{t+1},{\hat{y}}_{k}^{t+1}\right)-F_{k}\left(\theta^{t},{\hat{y}}_{k}^{t+1}\right)\leq \langle \nabla_{\theta }F_{k}\left(\theta^{t},{\hat{y}}_{k}^{t+1}\right),\theta^{t+1}-\theta^{t}\rangle +\frac{L}{2}{∥\theta^{t+1}-\theta^{t}∥}^{2}. \end{array}$$

According to (1a), we have

(A4) $$\begin{array}{c} F_{k}\left(\theta^{t},{\hat{y}}_{k}^{t+1}\right)-F_{k}\left(\theta^{t},{\hat{y}}_{k}^{t}\right)=\alpha_{0}\left[L_{\mathrm{CE}}\left(\theta^{t};{\hat{y}}_{k}^{t+1}\right)-L_{\mathrm{CE}}\left(\theta^{t};{\hat{y}}_{k}^{t}\right)\right]+\alpha_{1}\left[r_{1}\left({\hat{y}}_{k}^{t+1}\right)-r_{1}\left({\hat{y}}_{k}^{t}\right)\right]. \end{array}$$

Since $L_{\mathrm{CE}}\left(\theta ;u_{k},{\hat{y}}_{k}\right)$ is a linear function with respect to ${\hat{y}}_{k}$, and $r_{1}\left({\hat{y}}_{k}\right)$ is a strongly convex function about ${\hat{y}}_{k}$, using Assumption 1, we have

(A5) $$\begin{array}{cc} & \alpha_{0}L_{\mathrm{CE}}\left(\theta^{t};u_{k},{\hat{y}}_{k}^{t}\right)+\alpha_{1}r_{1}\left({\hat{y}}_{k}^{t}\right)-\alpha_{0}L_{\mathrm{CE}}\left(\theta^{t};u_{k},{\hat{y}}_{k}^{t+1}\right)-\alpha_{1}r_{1}\left({\hat{y}}_{k}^{t+1}\right)\\ \; & \geq \langle \alpha_{0}\nabla_{y_{k}}L_{\mathrm{CE}}\left(\theta^{t};u_{k},{\hat{y}}_{k}^{t+1}\right)+\alpha_{1}\nabla_{y_{k}}r_{1}\left({\hat{y}}_{k}^{t+1}\right),{\hat{y}}_{k}^{t}-{\hat{y}}_{k}^{t+1}\rangle +\frac{\mu \alpha_{1}}{2}{∥{\hat{y}}_{k}^{t}-{\hat{y}}_{k}^{t+1}∥}^{2}. \end{array}$$

In (1), we see that ${\hat{y}}_{k}^{t+1}$ is the optimal solution. Using the first-order condition implies

(A6) $$\begin{array}{c} \langle \alpha_{0}\nabla_{y_{k}}L_{\mathrm{CE}}\left(\theta^{t};u_{k},{\hat{y}}_{k}^{t+1}\right)+\alpha_{1}\nabla_{y_{k}}r_{1}\left({\hat{y}}_{k}^{t+1}\right),{\hat{y}}_{k}^{t}-{\hat{y}}_{k}^{t+1}\rangle \geq 0. \end{array}$$

Substituting (A6) into (A5) and (A4) gives rise to

(A7) $$\begin{array}{c} F_{k}\left(\theta^{t},{\hat{y}}_{k}^{t+1}\right)-F_{k}\left(\theta^{t},{\hat{y}}_{k}^{t}\right))\leq -\frac{\mu \alpha_{1}}{2}{∥{\hat{y}}_{k}^{t}-{\hat{y}}_{k}^{t+1}∥}^{2}. \end{array}$$

Finally, we combine (A3) and (A4) to obtain the final result of this lemma by considering the relationship $F\left(\theta ,\hat{y}\right)=\frac{1}{K}\sum_{k=1}^{K}F_{k}\left(\theta ,{\hat{y}}_{k}\right)$ and taking the expected value. □

**Lemma** **A2.**
*Under Assumptions 1 and 2, it holds that*

(A8)
$$\begin{array}{cc} E[\langle \nabla_{\theta }F\left(\theta^{t},{\hat{y}}^{t+1}\right),\theta^{t+1}-\theta^{t}\rangle ]\leq & -\frac{\eta Q}{2}E[∥\nabla_{\theta }F\left(\theta^{t},{\hat{y}}^{t+1}\right){∥}^{2}]+\frac{\eta L^{2}}{2K}\sum_{k,q}E[∥\theta^{t}-\theta_{k,q}^{t}{∥}^{2}]\\ \; & -\frac{\eta }{2K^{2}Q}E[∥\sum_{k,q}\nabla_{\theta }F_{k}\left(\theta_{k,q}^{t},{\hat{y}}_{k}^{t+1}\right){∥}^{2}], \end{array}$$

*where $\eta =\eta_{\theta }\eta_{g}$.*

**Proof.** In order to bound the above error, we need to introduce the following two σ-algebras as follows:

$$\begin{array}{cc} F^{t}: & =\sigma \left(\{\xi_{k,q}^{t},\zeta_{k,q}^{t},S^{r}:k\in \left[K\right],0\leq q\leq Q-1,0\leq t\leq t-1\}\right),\\ F_{k,q}^{t}: & =\sigma \left(F^{t}\cup S^{t}\cup \left\{\xi_{k,l}^{t},\zeta_{k,l}^{t}:l\leq q\right\}\cup \left\{\xi_{j,l}^{t},\zeta_{j,l}^{t}:j\leq k-1,l\leq Q-1\right\}\right). \end{array}$$

Thus, it holds that

$$\begin{array}{cc} E[\langle \nabla_{\theta }F\left(\theta^{t},{\hat{y}}^{t+1}\right),\theta^{t+1}-\theta^{t}\rangle ] & =E[\langle \nabla_{\theta }F\left(\theta^{t},{\hat{y}}^{t+1}\right),-\frac{\eta_{\theta }\eta_{g}}{M}\sum_{k,q}I_{k\in S^{t}}g_{k}\left(\theta_{k,q}^{t},{\hat{y}}_{k}^{t+1}\right)\rangle ]\\ & =-\frac{\eta_{\theta }\eta_{g}}{M}\sum_{k,q}E\left[E\left[\langle \nabla_{\theta }F\left(\theta^{t},{\hat{y}}^{t+1}\right),I_{k\in S^{t}}g_{k}\left(\theta_{k,q}^{t},{\hat{y}}_{k}^{t+1}\right)\rangle \right]|F_{k,q-1}^{t}\right] \end{array}$$

where the last equality is due to the tower property of conditional expectation. Since $I_{k\in S^{t}}$ and $g_{k}\left(\theta_{k,q-1}^{t},{\hat{y}}_{k}^{t+1}\right)$ are $F_{k,q-1}^{t}$-measurable, we have that

(A9) $$\begin{array}{cc} E[\langle \nabla_{\theta }F\left(\theta^{t},{\hat{y}}^{t+1}\right),\theta^{t+1}-\theta^{t}\rangle ] & =E[\langle \nabla_{\theta }F\left(\theta^{t},{\hat{y}}^{t+1}\right),-\frac{\eta_{\theta }\eta_{g}}{M}\sum_{k,q}I_{k\in S^{t}}g_{k}\left(\theta_{k,q}^{t},{\hat{y}}_{k}^{t+1}\right)\rangle ]\\ & =-\frac{\eta_{\theta }\eta_{g}}{M}\sum_{k,q}E\left[\langle \nabla_{\theta }F\left(\theta^{t},{\hat{y}}^{t+1}\right),I_{k\in S^{t}}E\left[g_{k}\left(\theta_{k,q}^{t},{\hat{y}}_{k}^{t+1}\right)\right]\rangle \right]\\ \; & =-\frac{\eta_{\theta }\eta_{g}}{K}\sum_{k,q}E\left[\langle \nabla_{\theta }F\left(\theta^{t},{\hat{y}}^{t+1}\right),\nabla_{\theta }F_{k}\left(\theta_{k,q}^{t},{\hat{y}}_{k}^{t+1}\right)\rangle \right], \end{array}$$

where the last equality is due to (11), and we sample clients $S^{t}$ uniformly randomly, which leads to $E\left[I_{k\in S^{t}}\right]=\frac{M}{K}$. Based on the common equality $-\langle a,b\rangle =\frac{1}{2}{[∥a-b∥}^{2}-{∥a∥}^{2}-{∥b∥}^{2}]$, we know

(A10) $$\begin{array}{cc} & E[\langle \nabla_{\theta }F\left(\theta^{t},{\hat{y}}^{t+1}\right),\theta^{t+1}-\theta^{t}\rangle ]\\ & =-\eta QE[\langle \nabla_{\theta }F\left(\theta^{t},{\hat{y}}^{t+1}\right),\sum_{k,q}\frac{1}{KQ}\nabla_{\theta }F_{k}\left(\theta_{k,q}^{t},{\hat{y}}_{k}^{t+1}\right)\rangle ]\\ & =-\frac{\eta Q}{2}\left[E[∥\nabla_{\theta }F\left(\theta^{t},{\hat{y}}^{t+1}\right){∥}^{2}]+E[∥\sum_{k,q}\frac{1}{KQ}\nabla_{\theta }F_{k}\left(\theta_{k,q}^{t},{\hat{y}}_{k}^{t+1}\right){∥}^{2}]\right]\\ \; & \;\;+\frac{\eta Q}{2}E[∥\nabla_{\theta }F\left(\theta^{t},{\hat{y}}^{t+1}\right)-\sum_{k,q}\frac{1}{KQ}\nabla_{\theta }F_{k}\left(\theta_{k,q}^{t},{\hat{y}}_{k}^{t+1}\right){∥}^{2}]. \end{array}$$

Note that

$$\begin{array}{c} \nabla_{\theta }F\left(\theta^{t},{\hat{y}}^{t+1}\right)=\sum_{k,q}\frac{1}{KQ}\nabla_{\theta }F_{k}\left(\theta^{t},{\hat{y}}_{k}^{t+1}\right), \end{array}$$

We get

(A11) $$\begin{array}{c} E[∥\nabla_{\theta }F\left(\theta^{t},{\hat{y}}^{t+1}\right)-\sum_{k,q}\frac{1}{KQ}\nabla_{\theta }F_{k}\left(\theta_{k,q-1}^{t},{\hat{y}}_{k}^{t+1}\right){∥}^{2}]\leq \frac{L^{2}}{KQ}\sum_{k,q}E[∥\theta^{t}-\theta_{k,q}^{t}{∥}^{2}], \end{array}$$

where the inequality is due to the convexity of ${∥\cdot ∥}^{2}$ and the *L*-smooth assumption (10). Substituting (A11) into (A10), we obtain the final result. □

**Lemma** **A3.**
*Let $\eta =\eta_{\theta }\eta_{g}$, under Assumptions 2–4, it holds that*

(A12)
$$\begin{array}{cc} & E[∥\theta^{t+1}-\theta^{t}{∥}^{2}]\\ & \leq \frac{\eta^{2}\left(K-M\right)}{MK(K-1)}\left(3KQ^{2}E[∥\nabla_{\theta }F\left(\theta^{t},{\hat{y}}^{t+1}\right){∥}^{2}]+3KQ^{2}\sigma_{G}^{2}+3QL^{2}\sum_{k,q}E[∥\theta^{t}-\theta_{k,q}^{t}{∥}^{2}]\right)\\ \; & \;\;+\frac{\eta^{2}\left(M-1\right)}{MK(K-1)}E[∥\sum_{k,q}\nabla_{\theta }F_{k}\left(\theta_{k,q}^{t},{\hat{y}}_{k}^{t+1}\right){∥}^{2}]+\frac{\eta^{2}Q\sigma_{\theta }^{2}}{M}. \end{array}$$

**Proof.** By using Assumption 3 and equation (14), we have

(A13) $$\begin{array}{cc} E[∥\theta^{t+1}-\theta^{t}{∥}^{2}] & =E[∥\frac{\eta }{M}\sum_{k,q}I_{k\in S^{t}}g_{k}\left(\theta_{k,q}^{t},{\hat{y}}_{k}^{t+1}\right){∥}^{2}]\\ \; & \leq \frac{\eta^{2}}{M^{2}}E[∥\sum_{k,q}I_{k\in S^{t}}\nabla_{\theta }F_{k}\left(\theta_{k,q}^{t},{\hat{y}}_{k}^{t+1}\right){∥}^{2}]+\frac{\eta^{2}Q\sigma_{\theta }^{2}}{M}. \end{array}$$

Let $t_{k}=\sum_{q=0}^{Q-1}\nabla_{\theta }F_{k}\left(\theta_{k,q}^{t},{\hat{y}}_{k}^{t+1}\right)$. Using this definition and expanding the squared norm, we have

(A14) $$\begin{array}{c} E[∥\sum_{k,q}I_{k\in S^{t}}\nabla_{\theta }F_{k}\left(\theta_{k,q}^{t},{\hat{y}}_{k}^{t+1}\right){∥}^{2}]=E[∥\sum_{k=1}^{K}I_{k\in S^{t}}t_{k}{∥}^{2}]=\sum_{i,j}E\left[I_{i\in S^{t}}I_{j\in S^{t}}\langle t_{i},t_{j}\rangle \right]. \end{array}$$

For the case where i=j, noting that $I_{i\in S^{t}}$ is an indicator function and $P\left(i\in S^{t}\right)=\frac{M}{K}$, we have

$$\begin{array}{c} E\left[I_{i\in S^{t}}I_{j\in S^{t}}\langle t_{i},t_{j}\rangle \right]=E\left[I_{i\in S^{t}}\right]∥t_{i}{∥}^{2}=\frac{M}{K}E[∥t_{i}{∥}^{2}]. \end{array}$$

For the case where i≠j, considering the probability of selecting two distinct clients from K total clients, we obtain

$$\begin{array}{c} E\left[I_{i\in S^{t}}I_{j\in S^{t}}\langle t_{i},t_{j}\rangle \right]=\frac{M(M-1)}{K(K-1)}E\left[\langle t_{i},t_{j}\rangle \right]. \end{array}$$

Then, we derive that

(A15) $$\begin{array}{cc} E[∥\sum_{k=1}^{K}I_{k\in S^{t}}t_{k}{∥}^{2}] & =\frac{M}{K}\sum_{i=1}^{K}{∥t_{i}∥}^{2}+\frac{M(M-1)}{K(K-1)}\sum_{i\neq j}\langle t_{i},t_{j}\rangle \\ \; & =\frac{M^{2}}{K}\sum_{i=1}^{K}∥t_{i}{∥}^{2}-\frac{M(M-1)}{2K(K-1)}\sum_{i\neq j}{∥t_{i}-t_{j}∥}^{2}, \end{array}$$

where the last equality is due to the fact that $\langle a,b\rangle =\frac{1}{2}{[∥a∥}^{2}+{∥b∥}^{2}{-∥a-b∥}^{2}]$. Similarity,

(A16) $$\begin{array}{c} E[∥\sum_{k=1}^{K}t_{k}{∥}^{2}]=K\sum_{k=1}^{K}E[∥t_{k}{∥}^{2}]-\frac{1}{2}\sum_{i\neq j}E[∥t_{i}-t_{j}{∥}^{2}]. \end{array}$$

Substituting (A16) into (A15) implies that

(A17) $$\begin{array}{c} E[∥\sum_{k=1}^{K}I_{k\in S^{t}}t_{k}{∥}^{2}]=\frac{KM-M^{2}}{K(K-1)}\sum_{i=1}^{K}∥t_{i}{∥}^{2}+\frac{M(M-1)}{K(K-1)}{∥\sum_{i=1}^{K}t_{i}∥}^{2}. \end{array}$$

By using Assumptions 2 and 4, we have

(A18) $$\begin{array}{cc} \sum_{k=1}^{K}E[∥t_{k}{∥}^{2}]= & \sum_{k=1}^{K}E[∥\sum_{q=0}^{Q-1}[\nabla_{\theta }F_{k}\left(\theta_{k,q}^{t},{\hat{y}}_{k}^{t+1}\right)-\nabla_{\theta }F_{k}\left(\theta^{t},{\hat{y}}_{k}^{t+1}\right)+\nabla_{\theta }F_{k}\left(\theta^{t},{\hat{y}}_{k}^{t+1}\right)\\ & -\nabla_{\theta }F\left(\theta^{t},{\hat{y}}^{t+1}\right)+\nabla_{\theta }F\left(\theta^{t},{\hat{y}}^{t+1}\right){]∥}^{2}]\\ \leq & 3QL^{2}\sum_{k,q}E[∥\theta^{t}-\theta_{k,q}^{t}{∥}^{2}]+3KQ^{2}\sigma_{G}^{2}+3KQ^{2}E[∥\nabla_{\theta }F\left(\theta^{t},{\hat{y}}^{t+1}\right){∥}^{2}]. \end{array}$$

Substituting (A17) and (A18) into (A13), we obtain the final result. □

**Lemma** **A4.**
*Under Assumptions 2–4, if $2Q\eta_{g}^{2}L^{2}\leq \frac{1}{Q-1}$, we have*

(A19)
$$\begin{array}{cc} & \sum_{k,q}E[∥\theta^{t}-\theta_{k,q}^{t}{∥}^{2}]\\ & \leq 4\left(Q-1\right)\left(4KQ^{2}\eta_{g}^{2}E[[∥\nabla_{\theta }F\left(\theta^{t},{\hat{y}}^{t+1}\right){∥}^{2}]+4KQ^{2}\eta_{g}^{2}\sigma_{G}^{2}+KQ\eta_{g}^{2}\sigma_{\theta }^{2}\right). \end{array}$$

**Proof.** For any worker k∈[K], we have

(A20) $$\begin{array}{cc} & E[∥\theta^{t}-\theta_{k,q}^{t}{∥}^{2}]=E[∥\theta^{t}-\theta_{k,q-1}^{t}+\eta_{g}g_{k}\left(\theta_{k,q-1}^{t},{\hat{y}}_{k}^{t+1}\right){∥}^{2}]\\ & \leq E[∥\theta^{t}-\theta_{k,q-1}^{t}+\eta_{g}F_{k}\left(\theta_{k,q-1}^{t},{\hat{y}}_{k}^{t+1}\right){∥}^{2}]+\eta_{g}^{2}\sigma_{\theta }^{2}\\ \; & \leq \left(1+\frac{1}{Q-1}\right)E[∥\theta^{t}-\theta_{k,q-1}^{t}{∥}^{2}]+Q\eta_{g}^{2}E[∥\nabla_{\theta }F_{k}\left(\theta_{k,q-1}^{t},{\hat{y}}_{k}^{t+1}\right){∥}^{2}]+\eta_{g}^{2}\sigma_{\theta }^{2}, \end{array}$$

where the first inequality is due to Assumption 3 and the second inequality follows from the inequality $2\langle a,b\rangle \leq {\kappa ∥a∥}^{2}+\frac{1}{\kappa }{∥b∥}^{2}$ and setting κ=Q−1. Furthermore,

(A21) $$\begin{array}{cc} & E[∥\nabla_{\theta }F_{k}\left(\theta_{k,q-1}^{t},{\hat{y}}_{k}^{t+1}\right){∥}^{2}]\\ & \leq 2E[∥\nabla_{\theta }F_{k}\left(\theta_{k,q-1}^{t},{\hat{y}}_{k}^{t+1}\right)-\nabla_{\theta }F_{k}\left(\theta^{t},{\hat{y}}_{k}^{t+1}\right){∥}^{2}]+2E[∥\nabla_{\theta }F_{k}\left(\theta^{t},{\hat{y}}_{k}^{t+1}\right){∥}^{2}]\\ \; & \leq 2L^{2}∥\theta^{t}-\theta_{k,q-1}^{t}{∥}^{2}+4E[∥\nabla_{\theta }F\left(\theta^{t},{\hat{y}}^{t+1}\right){∥}^{2}]+4E[∥\nabla_{\theta }F\left(\theta^{t},{\hat{y}}^{t+1}\right)-\nabla_{\theta }F_{k}\left(\theta^{t},{\hat{y}}_{k}^{t+1}\right){∥}^{2}], \end{array}$$

where the last inequality is due to Assumption 2. Substituting (A21) into (A20), with Assumption 4, we have

(A22) $$\begin{array}{cc} \sum_{k,q}E[∥\theta^{t}-\theta_{k,q}^{t}{∥}^{2}]\leq \left(1+\frac{1}{Q-1}+2Q\eta_{g}^{2}L^{2}\right)[ & \sum_{k,q}E[∥\theta^{t}-\theta_{k,q-1}^{t}{∥}^{2}]+4KQ^{2}\eta_{g}^{2}[∥\nabla_{\theta }F\left(\theta^{t},{\hat{y}}^{t+1}\right){∥}^{2}]\\ \; & +4KQ^{2}\eta_{g}^{2}\sigma_{G}^{2}+KQ\eta_{g}^{2}\sigma_{\theta }^{2}]. \end{array}$$

Consider $2Q\eta_{g}^{2}L^{2}\leq \frac{1}{Q-1}$; then, we have

(A23) $$\begin{array}{cc} \sum_{k,q}E[∥\theta^{t}-\theta_{k,q}^{t}{∥}^{2}]\leq \left(1+\frac{2}{Q-1}\right)[ & \sum_{k,q}E[∥\theta^{t}-\theta_{k,q-1}^{t}{∥}^{2}]+4KQ^{2}\eta_{g}^{2}[∥\nabla_{\theta }F\left(\theta^{t},{\hat{y}}^{t+1}\right){∥}^{2}]\\ \; & +4KQ^{2}\eta_{g}^{2}\sigma_{G}^{2}+KQ\eta_{g}^{2}\sigma_{\theta }^{2}]. \end{array}$$

Unrolling the recursion, we get

(A24) $$\begin{array}{c} \sum_{k,q}E[∥\theta^{t}-\theta_{k,q}^{t}{∥}^{2}]\leq \sum_{l=0}^{q}{\left(1+\frac{2}{Q-1}\right)}^{l}\left(4KQ^{2}\eta_{g}^{2}[∥\nabla_{\theta }F\left(\theta^{t},{\hat{y}}^{t+1}\right){∥}^{2}]+4KQ^{2}\eta_{g}^{2}\sigma_{G}^{2}+KQ\eta_{g}^{2}\sigma_{\theta }^{2}\right). \end{array}$$

Since $\sum_{l=0}^{q}{\left(1+\frac{2}{Q-1}\right)}^{l}\leq \frac{Q-1}{2}{\left(1+\frac{2}{Q-1}\right)}^{Q-1}\leq 4\left(Q-1\right)$ and 1+x≤exp(x), we have the final result. □

### Appendix A.2. The Proof of Theorem 1

Using Lemmas A1–A3, we have

$$\begin{array}{cc} \; & E\left[F\left(\theta^{t+1},{\hat{y}}^{t+1}\right)\right]-E\left[F\left(\theta^{t},{\hat{y}}^{t}\right)\right]\\ & \leq -\frac{\eta Q}{2}E[∥\nabla_{\theta }F\left(\theta^{t},{\hat{y}}^{t+1}\right){∥}^{2}]+\frac{\eta L^{2}}{2K}\sum_{k,q}E[∥\theta^{t}-\theta_{k,q}^{t}{∥}^{2}]-\frac{\eta }{2K^{2}Q}E[∥\sum_{k,q}\nabla_{\theta }F_{k}\left(\theta_{k,q}^{t},{\hat{y}}_{k}^{t+1}\right){∥}^{2}]\\ & \;+\frac{L\eta^{2}\left(K-M\right)}{2MK(K-1)}\left(3KQ^{2}E[∥\nabla_{\theta }F\left(\theta^{t},{\hat{y}}^{t+1}\right){∥}^{2}]+3KQ^{2}\sigma_{G}^{2}+3QL^{2}\sum_{k,q}E[∥\theta^{t}-\theta_{k,q}^{t}{∥}^{2}]\right)\\ & \;+\frac{\eta^{2}\left(M-1\right)L}{2MK(K-1)}E[∥\sum_{k,q}\nabla_{\theta }F_{k}\left(\theta_{k,q}^{t},{\hat{y}}_{k}^{t+1}\right){∥}^{2}]+\frac{L\eta^{2}Q\sigma_{\theta }^{2}}{2M}-\frac{\mu \alpha_{1}}{2K}\sum_{k=1}^{K}E[∥{\hat{y}}_{k}^{t}-{\hat{y}}_{k}^{t+1}{∥}^{2}]. \end{array}$$

When $\eta LK\leq \frac{M(K-1)}{K(M-1)}$, we know $\frac{\eta }{2K^{2}Q}-\frac{\eta^{2}L\left(M-1\right)}{2MK(K-1)}\geq 0$. It leads to

$$\begin{array}{cc} E\left[F\left(\theta^{t+1},{\hat{y}}^{t+1}\right)\right]-E\left[F\left(\theta^{t},{\hat{y}}^{t}\right)\right]\leq & -\left(\frac{\eta Q}{2}-\frac{L\eta^{2}\left(K-M\right)3Q^{2}}{2M(K-1)}\right)E[∥\nabla_{\theta }F\left(\theta^{t},{\hat{y}}^{t+1}\right){∥}^{2}]\\ \; & +\left(\frac{\eta L^{2}}{2K}+\frac{3QL^{3}\eta^{2}\left(K-M\right)}{2MK(K-1)}\right)\sum_{k,q}E[∥\theta^{t}-\theta_{k,q}^{t}{∥}^{2}]\\ \; & +\frac{3LQ^{2}\sigma_{G}^{2}\eta^{2}\left(K-M\right)}{2M(K-1)}+\frac{L\eta^{2}Q\sigma_{\theta }^{2}}{2M}-\frac{\mu \alpha_{1}}{2K}\sum_{k=1}^{K}E[∥{\hat{y}}_{k}^{t}-{\hat{y}}_{k}^{t+1}{∥}^{2}]. \end{array}$$

Substituting Lemma A4 into the above inequality, we have

$$\begin{array}{cc} \; & E\left[F\left(\theta^{t+1},{\hat{y}}^{t+1}\right)\right]-E\left[F\left(\theta^{t},{\hat{y}}^{t}\right)\right]\\ \; & \leq -\eta Q\left(\frac{1}{2}-8L^{2}\left(Q-1\right)Q\eta_{g}^{2}-\frac{\eta L(K-M)Q}{2M(K-1)}\left(3Q+48L^{2}\left(Q-1\right)Q^{2}\eta_{g}^{2}\right)\right)E[∥\nabla_{\theta }F\left(\theta^{t},{\hat{y}}^{t+1}\right){∥}^{2}]\\ \; & \;+\left(\eta L^{2}\left(Q^{2}\left(Q-1\right)\right)\eta_{g}^{2}+\frac{24Q^{4}L^{3}\eta^{2}\eta_{g}^{2}\left(K-M\right)}{M(K-1)}+\frac{3LQ^{2}\eta^{2}\left(K-M\right)}{2M(K-1)}\right)\sigma_{G}^{2}\\ \; & \;+\left(2\eta L^{2}\left(Q-1\right)Q\eta_{g}^{2}+\frac{6Q^{2}\left(Q-1\right)L^{3}\eta^{2}\eta_{g}^{2}\left(K-M\right)}{M(K-1)}+\frac{L\eta^{2}Q}{2M}\right)\sigma_{\theta }^{2}\\ \; & \;-\frac{\mu \alpha_{1}}{2K}\sum_{k=1}^{K}E[∥{\hat{y}}_{k}^{t}-{\hat{y}}_{k}^{t+1}{∥}^{2}]. \end{array}$$

When $\eta_{g}^{2}\leq \frac{1}{64Q\left(Q-1\right)L^{2}}$ and $\eta \leq \frac{M(K-1)}{\left(3+12Q)QL(K-M\right)}$, we have

$$\begin{array}{c} 8L^{2}\left(Q-1\right)Q\eta_{g}^{2}+\frac{\eta L(K-M)Q}{2M(K-1)}\left(3Q+48L^{2}\left(Q-1\right)Q^{2}\eta_{g}^{2}\right)\leq \frac{1}{4}. \end{array}$$

Let $\frac{\eta Q}{4}\leq \frac{\mu \alpha_{1}}{2K}$; we know

(A25) $$\begin{array}{cc} \frac{\eta Q}{4}g_{t}\leq & E\left[F\left(\theta^{t},{\hat{y}}^{t}\right)\right]-E\left[F\left(\theta^{t+1},{\hat{y}}^{t+1}\right)\right]+\eta \eta_{g}^{2}\left(L^{2}\left(Q^{2}\left(Q-1\right)\right)\sigma_{G}^{2}+2L^{2}\left(Q-1\right)Q\sigma_{\theta }^{2}\right)\\ & +\eta^{2}\eta_{g}^{2}\left(\frac{24Q^{4}L^{3}\left(K-M\right)}{M(K-1)}\sigma_{G}^{2}+\frac{6Q^{2}\left(Q-1\right)L^{3}\left(K-M\right)}{M(K-1)}\sigma_{\theta }^{2}\right)\\ \; & +\eta^{2}\left(\frac{3LQ^{2}\left(K-M\right)}{2M(K-1)}\sigma_{G}^{2}+\frac{LQ}{2M}\sigma_{\theta }^{2}\right). \end{array}$$

Summing the above inequality above from 1 to *T*, we obtain

$$\begin{array}{cc} \frac{1}{T}\sum_{t=0}^{T-1}g_{t} & \leq \frac{4(E\left[F\left(\theta^{0},{\hat{y}}^{0}\right)\right]-E\left[F\left(\theta^{T},{\hat{y}}^{T}\right)\right])}{\eta QT}+\eta_{g}^{2}B_{1}+4\eta \eta_{g}^{2}B_{2}+4\eta B_{3}. \end{array}$$

Since 0 is the lower bound for cross-entropy loss $F(\theta ,\hat{y})$, we obtain the final result.
